# Determining the feasibility of an online, media mediation program for parents to improve parent-child sexual health communication

**DOI:** 10.23860/jmle-2020-12-1-2

**Published:** 2020-04-28

**Authors:** Tracy M. Scull, Christina Malik, Elyse Keefe

**Affiliations:** innovation Research & Training, USA

**Keywords:** middle school, sexual health, media mediation, media literacy education, parent-adolescent communication

## Abstract

Media Aware Parent is an interactive, web-based program designed to equip parents to communicate with their adolescent child about sexual health and media by enhancing parental communication and media mediation skills, as well as provide them with medically-accurate sexual health knowledge. In a small feasibility study, 56 parents of 7th and 8th graders in the United States were randomly assigned to complete a prototype of Media Aware Parent or receive online resources about adolescent sexual health. Results indicated that after using the program, participants in the intervention group discussed more new sexual health topics with their child and also had stronger beliefs in the importance of parent-adolescent communication (PAC) about sexual health when compared to the control group.

## INTRODUCTION

Parents can have a significant positive influence on the sexual health of their adolescent children. Adolescents whose parents regularly talk with them about sex are less likely to engage in unprotected sex (Weinman et al., 2008) and more likely to talk to their parents in the future about sex-related issues (Martino et al., 2008). A large meta-analysis found a significant positive relationship between parent-adolescent communication (PAC) about sex and safe sexual behaviors among adolescents including contraceptive and condom use (Widman et al., 2016). While peer influence can normalize and promote sexual activity among adolescents (Bleakley et al., 2009), research suggests that PAC may redress inaccurate normative beliefs, thus delaying sexual debut and reducing unprotected sex (Whitaker & Miller, 2000). Furthermore, the positive effects are maximized when conversations take place early—prior to first sexual intercourse (Clawson & Reese-Weber, 2003)—and on a regular basis (Martino et al., 2008). Fostering effective PAC about sex and relationships is important given that about 40% of U.S. high school students have had sexual intercourse (Kann et al., 2018) and adolescents are at risk for acquiring sexually transmitted infections (Kann et al., 2018) and experiencing unintended pregnancies (Finer & Zolna, 2016).

### Barriers to parent-teen communication about sexual health

While the vast majority (90%) of parents report speaking to some degree with their teen child about the negative consequences of sex, more than half reported they did not discuss sexual topics such as how to obtain condoms and other birth control methods (Swain, Ackerman, & Ackerman, 2006). Furthermore, many young people report having not discussed any sexual topics with their parent (Widman et al., 2014), suggesting that even when parents feel that they are communicating with their teen child, these attempts may be ineffective. Parents may lack the knowledge, communication skills, and preparation necessary for engaging in in-depth conversations about sexual health with their adolescent child (DiIorio et al., 2003). A recent systematic review of PAC revealed that many parents do not have an adequate knowledge base about sexual health, which negatively impacts their ability to communicate with their children (Flores & Barroso, 2017). Parents who report being knowledgeable about sexual health, and comfortable with sexual health communication, discuss more topics with their child than parents who reported difficulties with such communication (Jerman & Constantine, 2010). Similarly, parents who have misinformation about sexual health (e.g., believe that contraceptive methods are less effective than they actually are) report being less likely to discuss contraception with their children (Swain et al., 2006). Another barrier to parents talking with their child about sex is a lack of communication skills (Jerman & Constantine, 2010; Whitaker et al., 1999). Importantly, PAC is known to be most effective at improving youth outcomes when parents are able to talk to their child in an open, honest, comfortable, and knowledgeable manner (Jerman & Constantine, 2010; Whitaker et al., 1999).

It is encouraging that programs designed to help parents talk with their children about sex can be effective in increasing PAC (Santa Maria et al., 2015; Widman et al., 2019), and positively impact adolescent health outcomes, such as increasing condom use (Widman et al., 2016; Widman et al., 2019). However, many of the programs that are available for parents require them to attend in-person sessions, often over several weeks, which can be a significant barrier for many families. Researchers have suggested that computer-based interventions may be useful in overcoming such barriers associated with existing programs that are available to parents (Santa Maria et al., 2015; Widman et al., 2019).

### Media influence on youth

Another factor affecting youth sexual health is the messaging in popular media; yet, programs designed for parents to promote adolescent sexual health do not often address media influence on adolescent sexual health. Media usage increases greatly as youth enter early adolescence (Rideout, Foehr, et al., 2010). On average, adolescents in the U.S. (ages 13-18 years-old) spend about nine hours a day using entertainment media (Rideout, 2015). Media have been shown to have a unique influence on youth risk behaviors above and beyond the influence of parents and peers (Scull, Kupersmidt, & Erausquin, 2014; Scull et al., 2009; Scull, Malik, & Kupersmidt, 2018). Of concern, media messages related to sex and relationships, including those targeted toward teens, are often inaccurate and unhealthy (e.g., Ward et al., 2016). Content analyses of a variety of types of entertainment media have consistently found that sex is commonly depicted, yet any risks and/or responsibilities associated with sex are often missing (see Ward et al., 2016). Further, researchers have hypothesized that media may function as a “super-peer,” encouraging adolescents to have sex at younger ages (Brown, Halpern, & L’Engle, 2005). Studies have found that exposure to sexual content in mainstream media is associated with permissive attitudes about uncommitted sexual behaviors, expectations about sex, higher perceived peer sexual activity, and sexual behaviors (for a review see Ward et al., 2014). Higher levels of exposure to sexual content in entertainment media (e.g., television programs) are predictive of subsequent sexual behavior in adolescents (Ward et al., 2016) and of teen pregnancy (Chandra et al., 2008).

### Parental media mediation

Research has examined the impact of parents as a protective factor against the potential unhealthy influence of media on adolescent health. Parental media mediation has been defined as “any strategy parents use to control, supervise, or interpret content” for their child (Warren, 2001). The term typically refers to three strategies of parent-child interactions related to media use: 1) discussions about media content (active mediation); 2) parental rules or monitoring of media use (restrictive mediation); or 3) co-viewing, or consuming media together (Nathanson, 2001). Media mediation, particularly active and restrictive mediation, has an important influence on youth media exposure and well-being (Buijzen & Valkenberg, 2005; Nathanson, 2001; Valkenburg et al., 2013). Active mediation is associated with increased media processing skills in children (Buijzen & Valkenberg, 2005; Nathanson, 2004; Valkenburg et al., 1999), and negative reinforcement of unhealthy media messages by parents is associated with a reduction in children’s expectancies about positive behavioral consequences of the risk behavior (Radanielina Hita, Kareklas, & Pinkleton, 2018).

An evaluation of a family-based online program designed to promote communication about media messages and substance use was shown to reduce alcohol and tobacco use behaviors in elementary school-aged youth (Scull, Kupersmidt, & Weatherholt, 2017). Similarly, media literacy education (MLE), which equips people to critically analyze media messages, has been shown to be an effective approach to adoelscent sexual health education and has the potential to attenuate negative impacts of media on youth sexual outcomes. Meta-analyses of MLE interventions have found positive effects on both media-related cognitions (e.g., decreased perceived realism of media messages) and health behavior-related outcomes (e.g., enhanced self-efficacy to engage in healthy behaviors), including those related to adolescent sexual health (Jeong, Cho, & Hwang, 2012; Vahedi, Sibalis, & Sutherland, 2018). While the link between media literacy skills and behavior is not fully understood, models such as the message interpretation processing model suggest that both affective and logical processing of media messages affect health decision-making (Austin, Chen, & Grube, 2006). Others have argued that the ability to design and produce media messages, thereby strengthening autonomy, is a key component to instilling critical literacy (Greene, 2013; Pangrazio, 2016). This research suggests that teaching youth to critically analyze, evaluate, and produce media messages may enhance their sexual health outcomes.

Despite the potential positive effects of media mediation and helping youth critically analyze media messages, many parents do not regularly engage in these behaviors, particularly with older children (Nikken & Jansz, 2006; Rideout et al., 2010; Vittrup, 2009). Research has found that parents often underestimate the influence of media on their own children and do not believe that talking with their child about media or restricting their child’s media use is important (Meirick et al., 2009; Rasmussen et al., 2016). For example, less than half of youth 8-18 years old report that their families have rules about watching television, and parents report discussing media less often than setting rules about media (Jordan et al., 2006; Rideout, Roberts, & Foehr, 2005). Parents are more likely to use media mediation strategies if they hold more skeptical attitudes about media (Mendoza, 2009; Nathanson, 2001). Furthermore, the relationship between parental attitudes about media and employing parental media mediation strategies is mediated by their attitudes about media mediation (i.e., those who believe active and restrictive mediation are beneficial are more likely to utilize such strategies; Rasmussen et al., 2016).

Using media messages as a platform to discuss potentially difficult sexual health topics (e.g., contraception, unplanned pregnancy, sexual assault) may also reduce some of the potential discomfort or embarrassment associated with conversations about these topics. Studies have found that teachers report MLE programs to be an easier means of delivering sexual health content than traditional school-based programs (Scull, Malik, & Kupersmidt, 2014; Tebes et al., 2007). To extend this, it may be that discussing media messages provides a more comfortable means for parents to approach the topic of sexual health with their children, by distancing the conversation from the personal lives of the parent or child, and refocusing the discussion on a third party (e.g., celebrities, fictional characters) and hypothetical situations. For example, parents who are trained in media mediation could start a conversation with their teen about unplanned pregnancy by talking about the lyrics to a song they hear on the radio that promotes unprotected sex. Adolescents tend to perceive media and choices about media as personal and outside of the authority of parents; thus, it is critical that parental media mediation be autonomy-supportive and open to increase the likelihood that values will be internalized (Valkenburg et al., 2013).

### Program description

Based upon this research, a prototype program was developed to bridge the gaps in resources available to parents to help prepare them to talk about sex, relationships, and media with their adolescent child. *Media Aware Parent* is a self-paced web-based program that provides parents of adolescents with 1) skills to discuss and monitor youth media exposure, 2) medically-accurate sexual health and relationship knowledge, and 3) practice in PAC methods, delivered through an interactive web-based application. It encourages key features of autonomy-supportive parenting by teaching parents how to communicate in a way that takes the child seriously, provides rationale for rules and decisions, and encourages the child’s input (Joussemet, Landry, & Koestner, 2008). *Media Aware Parent* was designed to help parents communicate their values and expectations about adolescent romantic relationships and sexual activity while providing fact-based medical information.

The program helps parents identify and distinguish their own values from facts and reality (e.g., prevalence of behaviors, medical facts), so they can provide more comprehensive information that both clarifies familial values and gives factual information to their child. Parents learn effective ways to discuss media messages with adolescents in order to enhance their media literacy skills and counter unhealthy media messages that promote risky sexual behaviors (e.g., unprotected sexual activity). Throughout the program, parents practice media literacy skills to identify sexual health content that is typically left out of media messages, such as contraceptive methods, unintended pregnancy, sexually transmitted infections (STIs), and elements of healthy relationships. They then learn how to utilize these skills to engage in media mediation, particularly related to sexual health with their adolescent child, in order to enhance the child’s media literacy skills. Each media example is accompanied by an audio recording of a parent-child conversation, in which the parent facilitates the child’s critical thinking about the message being communicated in the media example, also known as “media deconstructions.”

Some of the content in *Media Aware Parent* can be saved as parents complete the program to later be shared with their child(ren). This content includes audio of parent-child media deconstructions, videos about sexual health (e.g., condom demonstration), interactive activities (e.g., contraceptive methods explorer), and links to trusted resources (e.g., Centers for Disease Control) that contain medically-accurate, inclusive sexual health information.

The program was developed as part of two phases whereby a briefer prototype of the program with only three lessons was created first. Across these lessons, parents review the basics of adolescent development and learn about the pervasiveness of media messages, its influence on youth, basic media literacy skills, and general information about effective communication with adolescents, including media mediation practices. They explore the importance of communicating values related to sexual health to adolescents, and distinguishing these values from sexual health facts, covering specific sexual health topics, including teen pregnancy and medically accurate facts related to pregnancy prevention and contraceptive methods.

The program provides medically accurate information and facts about STIs and STI prevention and provides parents with skills to practice with their child to help them refuse unwanted sexual activity. The present research study presents an evaluation of the program prototype as part of a small feasibility study.

### Research hypotheses

Important predictors of effective parent-child sexual health communication behaviors include parent comfort, self-efficacy, and knowledge (DiIorio et al., 2000; Jaccard, Dittus, & Gorgon, 2000; Jerman & Constantine, 2010; Whitaker et al., 1999). *Media Aware Parent* provides parents with evidence of the importance for early and consistent communication with their child about sexual health in order to motivate parent-initiated communication and increase parents’ expectancies that such communication will have an impact on their child’s wellbeing. The program addresses parental discomfort and lack of self-efficacy by providing parents with knowledge they need to feel prepared to have these conversations with their young adolescent, as well as giving suggestions for initiating conversations and handling difficult youth questions. Thus, the first hypothesis proposes:

*H1:* Parents who use *Media Aware Parent* will report stronger beliefs about the importance of, comfort for, self-efficacy for, and more positive outcome expectancies for PAC about sexual health at posttest compared with parents who use the active control materials.

Research has shown that parental comfort with PAC about sex and relationships and accurate sexual health knowledge are related to more frequent PAC on these topics (Jerman & Constantine, 2010; Swain et al., 2006). *Media Aware Parent* provides parents with guided examples for initiating conversations and educational resources (e.g., videos, media examples) that parents can share with their child. In addition, it focuses on communication skills development, increasing motivation for sexual health conversations, filling gaps in knowledge, and novel use of media mediation as a way to make sexual health communication easier. Therefore, the second hypothesis is as follows:

*H2:* Parents who use *Media Aware Parent* will report engaging in PAC with their teen about more sexual health topics at posttest as compared with parents who use the control materials.

One goal of a media mediation program for parents is to increase the parent’s own critical thinking about media messages, so they can engage in active media mediation that includes teaching their adolescent child media literacy skills. MLE works to increase media message skepticism by enhancing critical thinking skills that are used to evaluate the truth and accuracy of the messages contained within (Brown & Bobkowski, 2011). In *Media Aware Parent*, parents learn the application of basic media literacy strategies, including learning to use critical thinking skills related to media and identifying the ways in which media messages are unrealistic. Therefore, the third hypothesis proposes:

*H3:* Parents who use *Media Aware Parent* will show higher levels of media message skepticism and decreased perceived realism of media messages at posttest as compared with parent who use the control materials.

## METHOD

### Participants

Sixty-five parents of 7^th^ and 8^th^ graders were randomly assigned to either the intervention (*n* = 33) or control group (*n* = 32) based on demographics (i.e., sex, race/ethnicity) for stratified sampling. Participants were recruited from across the United States. Three participants in the intervention group and two participants in the control group did not review their assigned sexual health materials and were dropped from the study. Two participants from each group did not complete the posttest. The final sample included 56 participants (control group *n* = 28; intervention group *n* = 28). The majority of participants (79%) identified as female (*n* = 44). Respondents self-reported their race and ethnicity using racial and ethnic categories and definitions designated for the United States National Institutes of Health (NIH) reporting. The racial breakdown of the sample was as follows: approximately 82% White or Caucasian (*n* = 46); 11% Black or African American (*n* = 6); and 7% Asian (*n* = 4). No participants reported their race as American Indian or Alaska Native, or Native Hawaiian or Other Pacific Islander. Approximately 3% of the sample identified themselves as Hispanic, Latino, or of Spanish descent (*n* = 2).

### Procedures

The methods and measures used in this study were approved by an Institutional Review Board (IRB). Parents were recruited through flyers and a recruitment website, which were developed by the research team, and were advertised on Craigslist, Facebook, and e-flyer services sent by schools to parents through email. Inclusion criteria stipulated that participants be the custodial parent or caregiver of a child in 7^th^ or 8^th^ grade. Participants were also required to be proficient in English. Once eligible participants reviewed and completed an online consent form, research staff phoned participants to give them a unique ID to be used in place of their name to ensure confidentiality for this study. Once recruitment was complete, participants were emailed a link to the web-based pretest questionnaire. After completion of the pretest, participants were emailed instructions on how to access their assigned materials, either 1) *Media Aware Parent* prototype or 2) the active control materials.

Participants randomly assigned to the active control group received online access to professionally designed materials from prominent health promotion organizations (e.g., Centers for Disease Control). Materials consisted of brochures (PDFs) and corresponded to topics in *Media Aware Parent* including adolescent sexuality, unplanned pregnancy, and STIs. The control program was designed to be consistent with the information that a parent may find when searching the internet for medically-accurate and developmentally appropriate sexual health information to share with their child. Control materials did not contain information about media literacy or media mediation.

Participants were emailed a link to the web-based posttest questionnaire about four weeks after completing their assigned program materials. Participants received a gift card incentive after completing each component of the study (i.e., $20 for pretest; $50 for resource review; $30 for posttest). The intervention and control groups took the pretest and posttest questionnaires 45 days apart, on average (intervention *M* = 45.58, *SD* = 3.00; control *M* = 45.75; *SD* = 2.73).

### Measures

Pretest-posttest questionnaires included measures of demographic background characteristics, communication outcomes, sexual health knowledge, media-related cognitions, and consumer satisfaction. Details on these measures, including psychometrics for scales can be found in Table 1. Participants were asked to reply to the questions with their 7^th^ or 8^th^ grade child in mind. If the participant had more than one child in those grades, they were asked to respond about the eldest of those children. This was included to avoid parents responding about multiple children and to reduce sampling bias (e.g., to prevent parents from choosing to answer about the child with whom they had better communication quality).

## RESULTS

### Preliminary analyses

Analyses first examined whether the randomization produced approximately equal samples on a number of demographic background variables. Control and intervention groups did not significantly differ from one another at pretest (*p* > .05) for chi-squared analyses in terms of participants’ gender, race, ethnicity, relationship status, and level of education (see Table 2).

T-tests were used to test for mean differences between the control and interventions groups on several other pretest demographic background variables and found no significant differences (*p* > .05). Participants in the control group were approximately the same age on average (*M* = 42.85 years, *SD*=6.44) to those in the intervention group (*M* = 43.82 years, *SD* = 5.86). The same can be said for their children (*M* = 12.82 years, *SD* = .90; *M* = 12.90 years, *SD* = .90). The control group reported a similarity level of religiosity on average (*M* = 2.67, *SD* = .94) to the intervention group (*M* = 2.45, *SD* = .82). Likewise, parents in the control group reported on average similar beliefs about the extent of their child’s previous romantic experience (*M* = 1.37, *SD* = .56) as parents in the intervention group (*M* = 1.34, *SD* = .48). Finally, parents in the control group reported, on average, similar quality of PAC (*M* = 3.94, *SD* = .48) as parents in the intervention group (*M* = 3.90, *SD* = .57).

Next, general linear model (GLM) analyses probed differences in sexual health knowledge after completing the intervention and control group content. As expected, both groups showed an increase in their average knowledge scores between pretest and posttest, with the control group increasing from 18.85 (*SD* = 2.53) to 19.57 (*SD* = 3.62) and the intervention group increasing from 19.46 (*SD* = 3.10) to 20.26 (*SD* = 2.07). No statistically significant differences were found for sexual health knowledge at posttest (*F* = 0.70, *p* > .05).

### Program dosage

Intervention participants spent an average time of 1 hr 40 min using *Media Aware Parent* (*SD* = 1 hr 9 min). Control participants spent an average time of 40 min using the control resources (*SD* = 44 min). Parents in the intervention group discussed an average of 1.82 media examples from *Media Aware Parent* with their child (*SD* = 2.02; range 0-7) in the month between the pretest and posttest. The most widely discussed media example involved a popular song that describes saying “no” to someone’s romantic advances. The activity that accompanied the media example explained several different ways to refuse someone’s advances with varying intensity (e.g., gently vs. forcefully) and how to adjust the language for different relationship circumstances (e.g., close relationships vs. acquaintances).

### Program satisfaction

Program satisfaction was evaluated by calculating descriptive statistics of *Media Aware Parent* ratings provided by parents in the intervention group using a 4-point Likert scale, with higher scores indicating higher levels of positive feedback. Program usability and satisfaction across five different constructs were high: 1) comprehensibility (*M* = 3.62; *SD* = .44), 2) structure/appearance (*M* = 3.38; *SD* = .50), 3) content (*M* = 3.41; *SD* = .52), 4) engagement (*M* = 3.27; *SD* = .64), and 5) usefulness (*M* = 3.56; *SD* = .53).

### Evaluation of hypotheses

GLM analyses were used to investigate H1 and H3 (i.e., differences in scaled constructs including parents’ beliefs about PAC about sexual health and media-related cognitions) using condition (intervention/control) as the independent variable. Pretest scores for each outcome were included as predictor variables; therefore, outcome variable means are reported as adjusted posttest scores. To examine increases in PAC behaviors about sexual health (H2), a change score was created for each participant by subtracting the number of communication topics discussed at posttest from the number discussed at pretest. This change score was analyzed using a t-test with condition as the independent variable.

See Table 3 for results related to H1. One significant finding supported H1. A large effect of the intervention was found for parents’ belief in the importance of PAC about sexual health (*d* = .74; *F* = 7.28, *p* < .01). Specifically, at posttest, parents who received *Media Aware Parent* were more likely to believe that PAC about sexual health is important as compared with parents in the control group. No significant differences between groups were found for comfort with PAC about sexual health, self-efficacy for PAC about sexual health, or outcomes expectancies for PAC about sexual health. However, it is worth noting a trend that self-efficacy approached significance with a meaningful effect size, whereby parents in the intervention group reported more self-efficacy than the control group.

H2 was supported in that a significant intervention effect was revealed for the number of new sexual health topics that parents communicated with their child about between pretest and posttest (*t* = 2.11; *p* < .05). Specifically, parents who received *Media Aware Parent* reported, on average, communicating over three times more new sexual health topics (*M* = 2.89; *SD* = 4.14) during this period as the control group parents did (*M* = 0.82; *SD* = 3.06).

See Table 3 for results related to H3. H3 was not supported as no significant differences were found at posttest between groups with respect to media-related cognitions. However, it is worth noting a trend that media skepticism approached significance with a meaningful effect size, whereby parents in the intervention group reported more skepticism about media messages than the control group.

## DISCUSSION

The purpose of this small study was to determine the feasibility of a web-based media mediation and communication skills program for parents. The results of this study provide initial evidence that a web-based program designed specifically for parents can increase PAC related to sexual health. Parents who used the *Media Aware Parent* prototype discussed more new sexual health topics with their child as compared to parents in the control group, who only received online information on adolescent sexual health. The program was effective in imparting equal amounts of factual sexual health knowledge to parents, as compared to the control group. However, parents who used *Media Aware Parent* showed more behavioral change regarding communication, reporting discussion of more new sexual health topics with their child. This suggests that while knowledge is important to enhance PAC about sexual health, factors beyond knowledge may be needed to motivate parents to engage in communication with their adolescent child. Given research that demonstrates the importance of comfort, self-efficacy, and communication skills for influencing parent-child sexual health communication, *Media Aware Parent* addressed many of these influences, going beyond filling gaps in knowledge, to providing opportunities to practice communication skills with their child, examples of modeling effective parent-child conversations, and evidence-based tips for effective parenting. This study also found that parents who completed the program increased their belief in the importance of communicating with their young adolescent about sexual health, which may have contributed to parents discussing more new sexual health topics with their child beyond sexual health knowledge. Future research should explore the factors that mediate the intervention effects of *Media Aware Parent* on the number of topics discussed.

The majority of parents in the intervention group utilized media mediation activities as a means of communicating with their child about sexual health, indicating discussions about media as a promising strategy for encouraging PAC, and a potential approach to improve youth media literacy skills. While not significant, the desirable and meaningful small-to-medium effect size on media skepticism suggests that the program has the potential to make parents more skeptical of media messages, which is associated with parents being more likely to engage in active media mediation. Future research using a larger sample should explore the impact of *Media Aware Parent* on both parent and child reports of parent media mediation, the impact of the program on parent and child media-related cognitions, such as media skepticism, and any impact on youth health behaviors. The most widely discussed media example focused on refusal skills and consent. This suggests that parents find this to be a particularly important topic to discuss compared to medical topics (e.g., how pregnancy happens) that parents may trust their child can simply read about. Therefore, parents may be interested in more resources for initiating these conversations, and strategies for helping their child build these skills.

Finally, *Media Aware Parent*, is a web-based, self-paced, and interactive intervention designed to overcome common barriers associated with most programs currently available for parents (e.g., those that require in-person attendance at sessions). Program usability and satisfaction across five different constructs were rated highly by parents (i.e., comprehensibility, structure/appearance, content, engagement, and usefulness). Given the positive impact that parent-child sexual health communication can have on youth outcomes, there is a need for interventions which target and engage parents to initiate sexual health conversations with their children early, often, and effectively (Martino et al., 2008; Shoop & Davidson, 1994; Weinman et al., 2008).

### Strengths and limitations

This study’s strengths included randomization of participants to condition. This allowed for causal inferences about the impact of the program prototype to be determined. Furthermore, an active control group was used to increase the rigor of the research. Results indicate that the randomization process was successful in that the intervention and control groups were equivalent on all assessed demographic background variables at baseline. Parents reported on their PAC behaviors, rather than reporting only on changes in beliefs and intentions for PAC.

This study also had some limitations. First, knowledge, beliefs, and behaviors were assessed at pretest and four weeks after participants completed the program, while it is possible that these effects may continue to change over time. For example, parents may have been motivated to initiate conversations after completing the program, but this study did not assess whether these conversations were maintained in the long term.

Second, measures were self-reported by parents, but several studies have shown that parents and children report different behaviors, including those related to media mediation (Gentile et al. 2012). Since children’s reports of PAC behaviors may differ from those of parents, it is important for future studies to include a youth assessment to obtain a more accurate measure of the quality and frequency of parent communication. A youth assessment is also important in order to capture the knowledge, beliefs, media literacy skills, and sexual behaviors of the youth, themselves, whose outcomes are the primary target of interventions such as *Media Aware Parent*.

Third, this study found that the program increased the number of new sexual health topics parents reported discussing with their teen child. However, this study did not investigate the *quality* of the communication that took place. Research has shown that communication quality impacts the efficacy of PAC about sexual health, with research indicating open, honest, informal, comfortable, and knowledgeable conversations to be most effective (Widman et al., 2016).

Fourth, the study used convenience sampling, which may have introduced bias into the study. For example, involved and motivated parents may have been more interested in participating in this study than parents who do not place as much importance on adolescent sexual health. Additionally, the sample included mostly mothers and was not highly diverse with respect to race or ethnicity, so results of this feasibility study may not be generalizable to all parents. Given the non-significant, but meaningful effect sizes found for parents’ self-efficacy for parent-child sexual health communication and parents’ media skepticism, future research should examine whether *Media Aware Parent* affects these particular outcomes compared to parents who receive no materials, or to examine if a study with a larger sample will reveal statistically significant changes in those outcomes. Since the development of *Media Aware Parent*, a meta-analysis of parent-based programs found that programs were most effective when they targeted both parents and youth, particularly younger adolescents, were designed to be culturally relevant, and required 10 hours or more of time on task (Widman et al., 2019). Therefore, while the program fulfills some of these features, it could benefit from being adapted for particular target groups and adding additional content in light of these new findings. Also important to note, the study took place in the United States with participants proficient in English. Future studies should explore and evaluate cultural adaptations of the program.

## Conclusion

The findings from this study suggest that a parent resource with an MLE focus may be a promising approach to promote PAC about sexual health. Providing medically-accurate sexual health information alone may not be enough to increase the frequency of parent-initiated communication behavior about sexual health with their children. Instead, parents may need to also be provided with the skills to initiate open, comfortable, and knowledgeable conversations.

## Figures and Tables

**Table 1.** Measures included on the feasibility pretest and posttest questionnaires with psychometrics for scaled constructs

Construct	# items	Response choices	Sample item	α
Demographic background				
Religiosity^a^	6	4pt. (Strongly disagree to Strongly agree)	My faith involves all of my life.	0.95
Beliefs about child’s romantic experience^b^	5	4pt. (Strongly disagree to Strongly agree)	How likely is it that your child has held hands with a boy/girl with romantic intent?	0.81
Quality of PAC^c^	20	5pt. (Strongly disagree to Strongly agree)	If my child were in trouble, she/he could tell me.	0.86
PAC about sexual health				
Behaviors^d^	25	Yes; No; Didn’t discuss but gave info	Have you ever talked to your child about what makes a relationship healthy?	--
Importance^d^	25	4pt. (Not important at all to Very important)	How important is it that you talk to your child about how to choose a method of birth control.	0.92
Comfort^d^	25	5pt. (Not comfortable at all to Very comfortable)	How comfortable do you feel discussing how to use a condom with your child?	0.97
Self-efficacy^d^	25	7pt. (Not sure at all to Completely sure)	I can always explain to my child about how pregnancy happens.	0.98
Outcome expectancies^e^	23	5pt. (Strongly disagree to Strongly agree)	If I talk with my child about sex topics, I think it will do some good.	0.88
Sexual health knowledge	11	Multiple choice; True/False	Condoms provide full protection against HPV.	--
Media-related cognitions				
Perceived realism^f^	6	4pt. (Strongly disagree to Strongly agree)	Teens in the media do things that average teens do.	0.84
Media skepticism^f^	5	4pt. (Strongly disagree to Strongly agree)	The media are dishonest about what happens when people drink alcohol.	0.78
Discussion of media examples (intervention)	11	Yes; No	Discussed media message: *The Fosters* TV clip.	--
Satisfaction with *MAP* (intervention)				
Comprehension^g^	3	4pt. (Strongly disagree to Strongly agree)	I found the information presented easy to understand.	0.93
Structure/Appearance^g^	3	4pt. (Strongly disagree to Strongly agree)	I found it easy to navigate through the resource.	0.68
Content^g^	2	4pt. (Strongly disagree to Strongly agree)	The content in this resource was interesting.	--
Engagement^g^	3	4pt. (Strongly disagree to Strongly agree)	I enjoyed using the resource.	0.83
Usefulness^g^	2	4pt. (Strongly disagree to Strongly agree)	This program could help me talk to my child	--

a Adapted from Hoge (1972)

b Adapted from Harris (2009)

c Adapted from Prado (2007)

d Adapted from Schuster et al. (2008)

e Adapted from Dilorio et al. (2000)

f Adapted from Kupersmidt, Scull, & Austin (2010)

g Adapted from Scull et al. (2016)

**Table 2.** Background characteristics of the sample by condition

Demographic Characteristics	Control (*n* = 28)	Intervention (*n* = 28)
Gender		
Female	82%	75%
Ethnicity		
Not Elispanic	93%	100%
Race		
Asian	7%	7%
Black	14%	7%
White	78%	86%
Relationship status		
Single/divorced/separated	21%	18%
Married/partnered	79%	82%
Education		
2-year degree or less	21%	25%
4-year degree	32%	36%
Advanced degree	46%	39%

**Table 3.** Results from hypotheses testing (H1 and H3)

Measure	*Intervention* *M(SE)*	*Control* *M(SE)*	*F-* *stat*	*p-value*	*d*
H1: PAC about sexual health					
Importance	3.63 (.06)	3.40 (06)	7.28*	0.01	0.74
Comfort	3.76 (.08)	3.62 (.08)	1.42	0.24	
Self-efficacy	5.13 (.16)	4.83 (.15)	1.83	0.18	0.37
Outcome expectancies	3.79 (.05)	3.82 (.05)	0.26	0.61	
H3: Media-related cognitions					
Perceived realism	1.95 (.08)	1.86 (.08)	0.63	0.43	
Media skepticism	3.42 (.08)	3.27 (.08)	1.77	0.19	0.37

Note: Effect sizes were calculated for findings that were significant or approaching significance.

## Data Availability

All relevant data are within the paper and its Supporting Information files.
